# *Leishmania mexicana* Trypanothione Reductase Inhibitors: Computational and Biological Studies

**DOI:** 10.3390/molecules24183216

**Published:** 2019-09-04

**Authors:** Félix Matadamas-Martínez, Alicia Hernández-Campos, Alfredo Téllez-Valencia, Alejandra Vázquez-Raygoza, Sandra Comparán-Alarcón, Lilián Yépez-Mulia, Rafael Castillo

**Affiliations:** 1 Departamento de Farmacia, Facultad de Química, Universidad Nacional Autónoma de México, Mexico City 04510, Mexico; 2 Unidad de Investigación Médica en Enfermedades Infecciosas y Parasitarias, Unidad Médica de Alta Especialidad-Hospital de Pediatría, Centro Médico Nacional Siglo XXI, Instituto Mexicano del Seguro Social, Mexico City 06720, Mexico; 3 Facultad de Medicina y Nutrición, Universidad Juárez del Estado de Durango Av. Universidad y Fanny Anitúa S/N, Durango 34000, Mexico

**Keywords:** trypanothione reductase, molecular docking, enzyme inhibitors, leishmanicidal activity

## Abstract

Leishmanicidal drugs have many side effects, and drug resistance to all of them has been documented. Therefore, the development of new drugs and the identification of novel therapeutic targets are urgently needed. *Leishmania mexicana* trypanothione reductase (LmTR), a NADPH-dependent flavoprotein oxidoreductase important to thiol metabolism, is essential for parasite viability. Its absence in the mammalian host makes this enzyme an attractive target for the development of new anti-*Leishmania* drugs. Herein, a tridimensional model of LmTR was constructed and the molecular docking of 20 molecules from a ZINC database was performed. Five compounds (ZINC04684558, ZINC09642432, ZINC12151998, ZINC14970552, and ZINC11841871) were selected (docking scores −10.27 kcal/mol to −5.29 kcal/mol and structurally different) and evaluated against recombinant LmTR (rLmTR) and *L. mexicana* promastigote. Additionally, molecular dynamics simulation of LmTR-selected compound complexes was achieved. The five selected compounds inhibited rLmTR activity in the range of 32.9% to 40.1%. The binding of selected compounds to LmTR involving different hydrogen bonds with distinct residues of the molecule monomers A and B is described. Compound ZINC12151998 (docking score −10.27 kcal/mol) inhibited 32.9% the enzyme activity (100 µM) and showed the highest leishmanicidal activity (IC_50_ = 58 µM) of all the selected compounds. It was more active than glucantime, and although its half-maximal cytotoxicity concentration (CC_50_ = 53 µM) was higher than that of the other four compounds, it was less cytotoxic than amphotericin B. Therefore, compound ZINC12151998 provides a promising starting point for a hit-to-lead process in our search for new anti-*Leishmania* drugs that are more potent and less cytotoxic.

## 1. Introduction

Leishmaniasis is one of the most diffused, neglected vector-borne diseases and causes 60,000 deaths annually [1]. Leishmaniasis is a poverty-associated disease. Different *Leishmania* species are responsible for the different clinical manifestations of the disease: (i) visceral, often known as kala-azar and the most serious form of the disease (*L. donovani* and *L. infantum/L. chagasi*); (ii) cutaneous, the most common (*L. major*, *L. tropica*, and *L. mexicana*); and (iii) mucocutaneous (*L. braziliensis*).

Pentavalent antimonials such as pentostam (sodium stibogluconate) and glucantime (*N*-methylglucamine antimonate) are used as first-line drugs for the treatment of leishmaniasis. Glucantime is proposed to inhibit trypanothione reductase (TR), and its genotoxic and mutagenic effects have been reported [2,3]. Amphotericin B and miltefosine are alternative drugs for the treatment of visceral (VL) and cutaneous leishmaniasis. All of them have diverse chemical origins and act via different modes of action [4]. Unfortunately, leishmaniasis treatment is still unsatisfactory, mainly due to the side effects leishmanicidal drugs produce, the long periods of treatment and high concentrations, and the high costs of treatment. Importantly, emergence of parasite resistance to the current leishmanicidal drugs has been reported [4].

Therefore, the development of new leishmanicidal drugs and the identification of novel parasite therapeutic targets are required. Different molecules involved in parasite survival and proliferation such as glycerol-3-phosphate dehydrogenase, hexokinase (glycolytic pathway), arginase, ornithine decarboxylase (polyamines pathway), trypanothione reductase, and glyoxalase (detoxification pathway) have been proposed as therapeutic targets [4,5]. 

Recently, new *N*-benzyl-1*H*-benzimidazol-2-amine derivatives have been synthesized, and one of them, named compound **8**, showed higher activity than miltefosine against the promastigote and amastigote of *L. mexicana* and *L. brasiliensis.* Interestingly, compound **8** inhibited 68.27% of the activity of recombinant *L. mexicana* arginase [6]. 

Herein, we report our search for new drugs against *L. mexicana* trypanothione reductase (LmTR). This enzyme is a homodimer NADPH-dependent flavoprotein oxidoreductase that participates in the parasite thiol metabolism [7]. LmTR reduces trypanothione disulfide T[S]_2_ to trypanotione T[SH]_2_, a low-molecular-mass dithiol. Trypanothione participates in central thiol–disulfide exchange reactions and participates as an electron donor in different metabolic pathways, from synthesis of DNA precursors to oxidant detoxification [8]. Trypanothione neutralizes the hydrogen peroxide produced by macrophages during infection through the tryparedoxin/tryparedoxin peroxidase I (TXN/TXNPx) system [8]. Therefore, TR inhibition increases the intracellular levels of reactive oxygen species lethal for the parasite. Besides this, the absence of the trypanothione redox system in mammals makes TR an attractive target for novel drug candidates [1].

To date, the crystal structure of the TR from *Trypanosoma cruzi* and *T. brucei* (TcTR and TbTR, respectively) and *L. infantum* (LiTR) has been obtained, and several molecules have been explored as enzyme inhibitors. Studies by high-throughput screening of an in-house library demonstrated that quinoline-based compounds and pyrimidopyridazine-based scaffolds have inhibitory activity against recombinant TbTR [9] and that lunarine analogs inhibit the activity of TcTR [10]. Clomipramine and its analogs are strong inhibitors of TcTR activity; however, its psychotropic activity precludes its use as an anti-trypanosomal drug [11]. 

In relation to LiTR, high-throughput screening studies identified novel 2-iminobenzimidazoles as enzyme inhibitors [12]. In addition, metals, metal-containing compounds, and antimony-containing drugs have been reported to inhibit LiTR [13,14,15]. Even more, in-house library and whole-cell screening assays have demonstrated the activity of diaryl sulfide, azole-based compounds, and polyamine analogs against LiTR [16,17]. For *L. braziliensis* TR, it was shown that it is the target of resveratrol analogues, and their leishmanicidal effect in vitro was demonstrated [18]. 

With the aim to identify LmTR inhibitors and considering that the LmTR crystal structure is unresolved, we herein report the tridimensional structure of LmTR determined from its amino acid sequence and by a homology approximation model. The SWISS-MODEL was selected for further refinement and construction of the homodimer structure. A subset of 20 druglike molecules with high affinity to LiTR (docking scores of −11.6 kcal/mol to −12.7 kcal/mol) obtained by virtual screening of 600,000 ZINC compounds was previously reported [19]. These molecules were submitted to a docking study focused on the catalytic site of the LmTR tridimensional structure, including NADPH and FAD domains. Five compounds were selected based on a broad range of docking scores (from −10.27 kcal/mol to −5.29 kcal/mol), as well as their structural diversity, to evaluate their in vitro inhibition of recombinant LmTR (rLmTR)activity and their leishmanicidal activity. In addition, the molecular dynamics interactions of LmTR-selected compounds are also described. 

## 2. Results and Discussion

### 2.1. Homology Modeling

**Figure 1:**
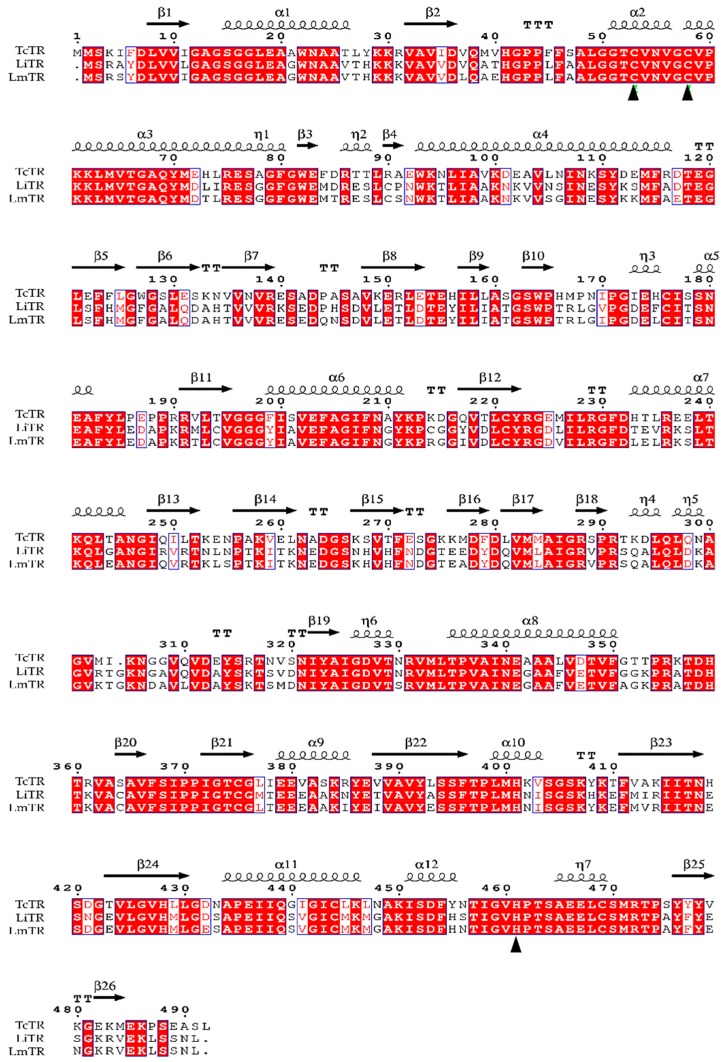
Multiple sequence alignment of trypanotione reductase from *L. infantum*, *L. mexicana*, and *Trypanosoma cruzi*. The LmTR sequence (XP_003871925.1) was aligned with homologous TR from *T. cruzi* (CAA78360.1) and *L. infantum* (XP_001462998.1) with a crystallographic structure reported in the PDB. Identical (conserved) amino acids in different sequences are marked with red-filled boxes, and the catalytic site (Cys52, Cys57, and His461) is shown with a triangle (▲). The alignment was performed in the Clustal-W program and ENDscript server.

The amino acid sequence of LmTR (XP_003871925.1) was obtained from NCBI (www.ncbi.nlm.nih.gov) and used to align with homologous proteins LiTR (XP_001462998.1) and TcTR (CAA78360.1) using the Clustal-W program and ENDscript server to carry out a subsequent selection of the best template (Figure 1).

Amino acid sequence alignment showed that LmTR shares 45% and 91% identity with TcTR and LiTR, respectively. LmTR is a dimeric protein where each monomer is formed by three domains: the FAD-binding domain (residues 1–160 and 289–360), the NADPH-binding domain (residues 161–288), and the interface domain (residues 361–488). The enzyme has one active site in each subunit; it includes the catalytic residues Cys52 and Cys57 from one monomer and His461 from the other monomer. The catalytic site and the NADPH and FAD domains are conserved in LmTR and LiTR, as well as in TcTR. 

The crystal structure of LiTR, PDB code 2JK6 [14], was selected as the template for the homology modeling protocol. The 3D model of LmTR was built using the SWISS-MODEL, I-Tasser, and Phyre2 servers (Table 1). The construction of a 3D model for LmTR was necessary because no crystallographic structure is available for this protein. 

The stereochemical quality of the three models was evaluated through the structure assessment tool on the WHATCHECK server. The SWISS-MODEL was selected based on the parameters of RMSD, Q-MEAN, and Ramachandran plot [20,21]. According to the Ramachandran plot, 96.4% of the residues were in the most favorable zones and 99.7% were in allowed regions (Table 1). Furthermore, the model obtained a QMEAN4 score of −0.17 between the model and the protein used as a template (values closer to 0 are preferred). The calculated RMSD value was 0.063 (lower values are indicative of stereochemical similarity between the template and target structures).

**Figure 2:**
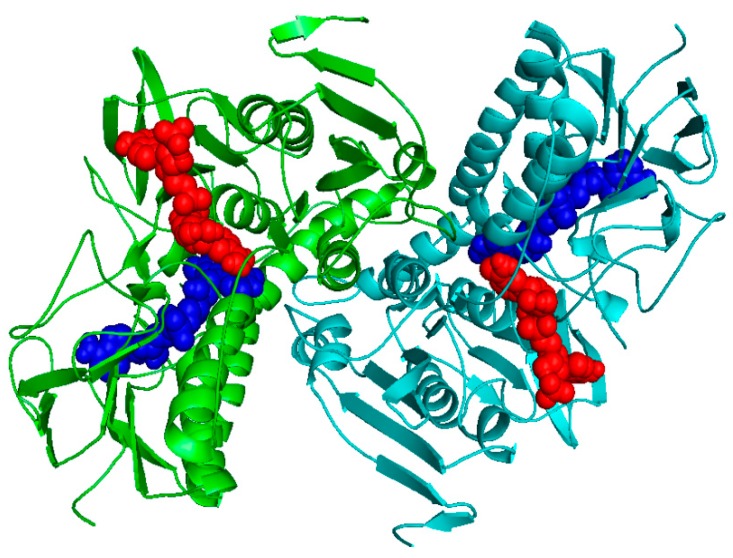
3D model of the LmTR built with the SWISS-MODEL server. The two subunits are colored in green and cyan. The FAD and NADPH are indicated using blue and red spheres, respectively. The picture was generated in PyMol [22].

The SWISS-MODEL was selected for further refinement and construction of the homodimer structure including NADPH and FAD according to crystallographic symmetry reports of LiTR (Figure 2).

### 2.2. Docking Studies 

The LmTR model generated was validated using the substrate trypanothione disulfide (T[S]_2_). In this, LmTR amino acids that interact with the substrate were the same as previously reported for LiTR and TcTR, determined by crystallographic studies [17,23]. The docking score obtained for the LmTR–T[S]_2_ complex was −5.32 kcal/mol; a similar docking score (−5.4 kcal/mol) was reported for TcTR [24]. In both studies, the AutoDock 4.2 program was used. Since the active sites of TR in these parasites are highly conserved, these assays validated the LmTR model generated.

In addition, the docking score of the TcTR inhibitor clomipramine was calculated using the generated LmTR model. In this case, a docking score of −5.5 kcal/mol was determined, while Pandey et al. [25], using the same inhibitor, calculated a docking score of −4.9 kcal/mol for LdTR. The differences in docking scores could be explained by the authors using another program (Maestro Glide program).

Twenty druglike molecules previously reported to have high affinity to LiTR [19] were submitted to a docking study focused on the catalytic site of the LmTR, and the docking score for each compound was selected considering their highest cluster size. The 20 molecules were found to bind selectively to the catalytic site of LmTR with docking scores lower than −4.8 kcal/mol.

**Figure 3:**
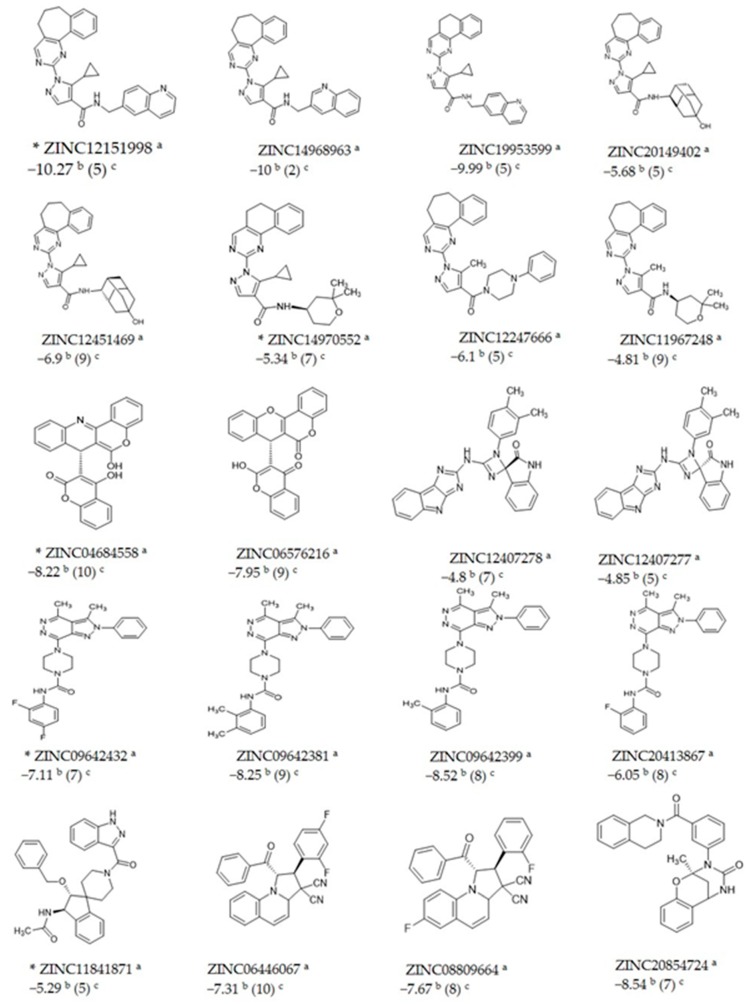
Docking scores (kcal/mol) and cluster sizes of compounds obtained with the AutoDock 4.2 program. * Compounds selected to test their inhibitory activity against rLmTR. ^a^ ZINC code, ^b^ ΔG bind (kcal/mol), ^c^ Cluster size.

The ZINC codes of the 20 structures and their estimated docking scores are listed in Figure 3. Compounds ZINC04684558, ZINC09642432, ZINC12151998, ZINC14970552, and ZINC11841871 were selected based on a broad range of docking scores (from −10.27 kcal/mol to −5.29 kcal/mol), as well as their structural diversity, to evaluate their in vitro inhibition of rLmTR activity and leishmanicidal activity. In addition, they were also selected considering their commercial availability.

In Figure 3, the structural similarity or diversity of the five selected compounds among themselves and the other ZINC compounds can be observed. Four of the five selected compounds are carboxamides, but they are structurally different. In a general description, ZINC09642432 is a pyridazine carboxamide; ZINC12151998 is an *N*-(6-quinolinemethyl)-3-pyrazole carboxamide; ZINC14970552 is an *N*-(2,2-dimethyltetrahydropiran-4-yl)-4-pyrazole carboxamide; and ZINC11841871 is a 3-indazole carboxamide. ZINC04684558 is not a carboxamide; it is a quinoline fused to a cumarine.

### 2.3. Expression and Purification of rLmTR

**Figure 4:**
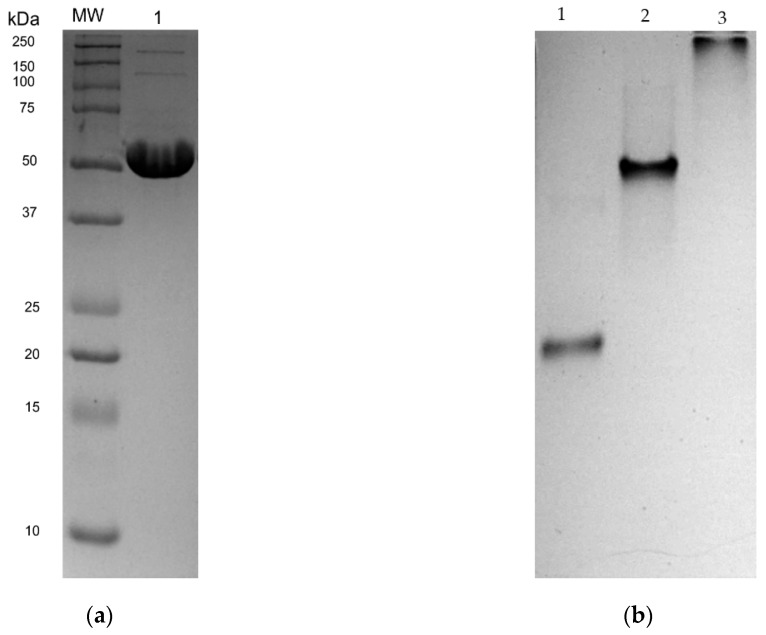
Electrophoretic analysis of rLmTR under reducing and native conditions: (**a**) 12% SDS-PAGE of affinity purified rLmTR (lane 1); MW: molecular weight markers (Precision Plus Protein Dual Color, BioRad); (**b**) 7.5% acrylamide native gel electrophoresis of affinity purified rLmTR (lane 2); bovine serum albumin (lane 1) and lactate dehydrogenase (lane 3) were included as markers.

The LmTR-ORF was subcloned in EcoRI and NotI sites in a pET28a expression vector. Sequencing of the expression construct pET28a-LmTR confirmed that the gene was in frame with a His tag at the N-terminal. rLmTR efficiently expressed by transformed BL21 (DE3) pLysS cells was purified by affinity column using 250 mM imidazole and analyzed in reducing (Figure 4a) and native conditions (Figure 4b). Bands of ~56 kDa in SDS-PAGE (Figure 4a lane 3) and 112 kDa in native-PAGE (Figure 4b, lane 2) were observed, corresponding to the monomer and homodimer of LmTR, respectively. 

### 2.4. Kinetic Characterization of rLmTR

**Figure 5:**
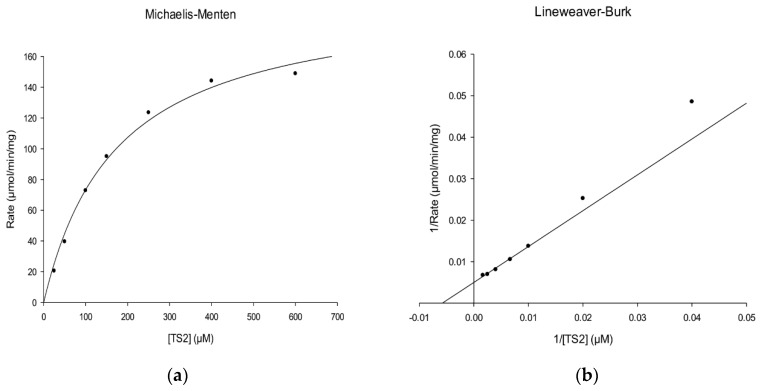
Kinetic characterization of rLmTR. (**a**) Michaelis–Menten and (**b**) Lineweaver–Burk double reciprocal plots.

The kinetic parameters of rLmTR followed the Michaelis–Menten equation with the substrate T[S]_2_. The Km and Vmax values of rLmTR using T[S]_2_ as a substrate were determined to be 173 µM (Figure 5a) and 200 µmol/min/mg (Figure 5b), respectively. The Km value of LmTR was slightly higher than that of LiTR (72 µM) [15]. The Vmax value was in the same order as that reported for LdTR (200 µmol/min/mg) [7].

### 2.5. rLmTR Inhibition Assays

Enzyme inhibition studies were performed with five selected compounds from the ZINC database. The inhibition results are presented in Table 2.

Compounds ZINC14970552, ZINC04684558, and ZINC09642432 (docking scores −5.34 kcal/mol, −8.22 kcal/mol, and −7.11 kcal/mol, respectively) inhibited 35.7%, 39.5%, and 40.1% of the activity of rLmTR when tested at 300 µM. On the other hand, compound ZINC11841871 (docking score −5.29 kcal/mol) achieved a similar inhibitory activity to compounds ZINC04684558 and ZINC09642432 when it was used at a lower concentration (150 µM).

Compound ZINC12151998 had a docking score of −10.27 kcal/mol and achieved inhibitory activity of 32.9%, slightly below the inhibitory activity obtained with compound ZINC14970552 (35.7%), although it was tested at lower concentration (100 µM vs. 300 µM). It is worth mentioning that compounds ZINC12151998 and ZINC11841871 showed inhibitory activity in the range obtained by the other compounds even when they were tested at 100 and 150 µM, respectively. Unfortunately, due to their poor solubility, their inhibitory activity at higher concentration could not be assessed.

### 2.6. Molecular Dynamics Simulation

**Figure 6:**
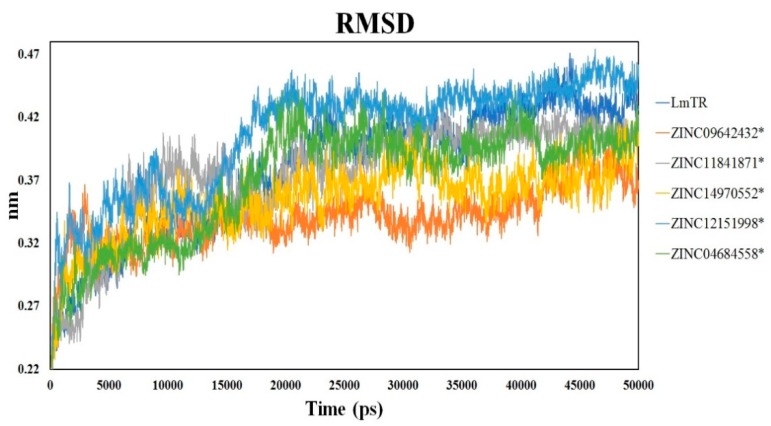
Root-mean-square deviation (RMSD) of LmTR and LmTR–inhibitor complexes. * denotes significant differences with respect to LmTR (*p* < 0.05). Statistical analysis was performed by applying ANOVA testing.

Molecular dynamics (MD) simulations of 50 ns were performed to characterize the interaction between LmTR and the five inhibitors. System stability was evaluated by means of the RMSD value. For each MD simulation, the first 17 ns were discarded from further analysis as the equilibration period. The six systems, LmTR (holoenzyme dimer with NADPH and FAD) and LmTR–inhibitor complexes, showed an RMSD value of 0.3 nm among Cα from initial to final structure conformation, showing that the system was stable throughout the simulation (Figure 6). It is worth highlighting that statistically significant differences (*p* < 0.05) were observed in the RMSD trajectory in each LmTR–inhibitor complex with respect to the holoenzyme, the highest being that of the LmTR–ZINC09642432 complex. These differences suggest that the binding of the compounds to LmTR provokes conformational changes.

The structural analysis showed that compounds bound in a different manner in both active sites (Figure S1). The hydrogen bonds formed for each inhibitor throughout the molecular dynamics simulations are presented in Table 3. Interestingly, at the end of the molecular dynamics simulations (50 ns), compound ZINC14970552 formed a hydrogen bond with A: Tyr198, a residue that participates in NADH binding [26], while compound ZINC04684558 made this interaction with B: Ser109, which is absent in human glutathione reductase and has been described as interacting with a competitive inhibitor of TcTR [27]. Additionally, compound ZINC04684558 formed a hydrogen bond with A: Glu466, a residue that in conjunction with His461 has been reported to participate in the binding of the auranofin thiosugar, a LiTR inhibitor [8]. Compound ZINC12151998 formed a hydrogen bond with B: Tyr110; this residue participates in interaction with diverse inhibitors reported for other parasitic TRs and has been described as a crucial amino acid for trypanothione disulfide binding [28]. It is worth mentioning that compounds ZINC04684558, ZINC11841871, and ZINC12151998 interacted throughout the molecular dynamics stimulation with amino acid residues important for the substrate binding, such as Glu18, Ser109, Glu466, Glu467, His461, Gly459, and Val58 [17,29].

**Figure 7:**
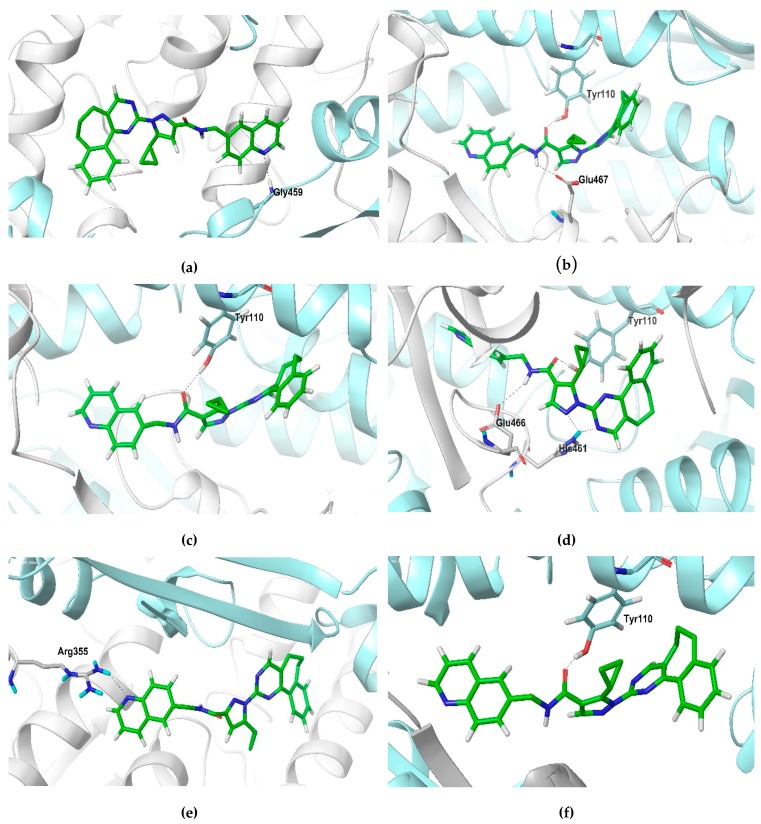
The hydrogen bonding interactions of compound ZINC12151998 with LmTR at 10 ns (**a**), 20 ns (**b**), 30 ns (**c**), 40 ns (**d**), and 50 ns (**e**,**f**). The LmTR monomers A and B are represented with gray ribbons and cyan ribbons, respectively. Carbon skeleton of compound ZINC12151998 is presented in green sticks, oxygen atoms are in red, nitrogen atoms are in blue, and hydrogen atoms are in grey. H-bonds are depicted as dotted lines.

The hydrogen bonding interactions of compound ZINC12151998 with LmTR protein throughout the molecular dynamics study are presented in Figure 7. (a) At 10 ns, a first interaction between the N of the quinoline nucleus and Gly459 at monomer B in Active Site 1 was achieved. (b) At 20 ns, interactions between the NH of the amide group and Glu467 at monomer A and the carbonyl group of the amide with the OH of Tyr110 at monomer B in Active Site 2 were observed. (c) At 30 ns, the interaction between the carbonyl group of the amide with de OH of Tyr110 at monomer B in Active Site 2 still remained. (d) At 40 ns, three main interactions were observed: the first one between the carbonyl group of the amide and the OH of Tyr110 at monomer B; the second one between the NH of the amide group and Glu466 at monomer A; and the third one a double interaction between Hist461 at monomer A and the N of pyrazole and the N of the pyrimidine nuclei, all of them in Active Site 2. (e, f) At 50 ns, two main interactions were observed: the N of the quinoline nucleus with Arg355 at monomer A in Active Site 1 and, once again, the carbonyl of the amide group with Tyr110 at monomer B in Active Site 2. 

Compound ZINC12151998 is an *N*-(6-quinolinemethyl)-3-pyrazole carboxamide, and molecular dynamics studies showed the importance of the quinoline nucleus in the formation of hydrogen bounds with the active site of LmTR. In addition, compound ZINC12151998 inhibited 32.9% of the activity of rLmTR. This shows that quinoline-based compounds have inhibitory activity against recombinant TbTR [9].

### 2.7. In Silico Analysis of ADME Properties

An important issue to address was the in silico prediction of the administration, distribution, metabolism, and excretion properties (ADME) and the possible toxicological effects of the five compounds tested in vitro (Table 4). The data demonstrated that the five compounds bear good physicochemical characteristics for consideration as potential drug candidates, such as the capacity to cross biological membranes and high gastrointestinal absorption, among others. Additionally, according to ADME analysis, the five compounds had drug likeness scores in the permitted range (values between −1 and 2 are accepted).

In addition, toxicological analysis revealed that their predicted LD_50_ values, estimated in rodents, was closer to 1–1.870 g/kg suggesting no potential toxicological effects with exception of ZINC12151998, that showed a LD_50_ of 500 mg/kg and was also predicted to be mutagenic; to confirm this profile, further in vitro and in vivo studies are required.

### 2.8. Leishmanicidal and Cytotoxic Activity

The activity of the selected compounds was evaluated against *L. mexicana* promastigotes. Amphotericin B, miltefosine, and glucantime were included as reference drugs. The results are presented in Table 5. All compounds were more active than glucantime (IC_50_ > 273.2 µM) but less active than amphotericin B (IC_50_ = 0.11 µM) and miltefosine (IC_50_ = 2.98 µM). Among the five tested compounds, ZINC12151998 showed the highest leishmanicidal activity (IC_50_ of 58 µM), followed by ZINC14970552 and ZINC11841871 (IC_50_ values of 147 and 159 µM, respectively). Compounds ZINC04684558 and ZINC09642432 had IC_50_ values above 200 µM. 

On the other hand, the cytotoxicity of all compounds was tested using the macrophage cell line J774.2; the results are presented in Table 5. The cytotoxicity of all compounds was lower than that of amphotericin B (CC_50_ = 7.4 µM), compounds ZINC04684558 and ZINC09642432 were less cytotoxic than glucantime (CC_50_ > 273.2 µM), and compounds ZINC04684558, ZINC09642432 and ZINC14970552 were less cytotoxic than miltefosine (CC_50_ = 141 µM). The cytotoxicity of compound ZINC12151998 was higher than that of the other four compounds (CC_50_ = 53 µM). The selectivity index (SI) values of the five compounds tested (0.6 to 1.5) indicated that they do not have specificity for the parasite. However, the same is true for glucantime (SI = 1.0). 

It is worth mentioning that compound ZINC12151998 showed the highest leishmanicidal activity among all the tested compounds. It showed affinity to the LmTR catalytic site with a docking score of −10.27 kcal/mol and it inhibited 32.9% of the activity of rLmTR (100 µM), similar to the inhibition percentages obtained by other compounds tested at higher concentrations. Compound ZINC12151998 is an *N*-(6-quinolinemethyl)-3-pyrazole carboxamide, and the participation of the quinoline nucleus in the formation of hydrogen bounds with the active site of LmTR was shown by molecular dynamics studies. These data agree with the inhibitory activity of quinoline-based compounds previously shown against recombinant TbTR [9]. 

## 3. Materials and Methods

### 3.1. General Information

Compounds ZINC04684558 and ZINC09642432 were purchased from ChemDiv (San Diego, CA, USA), and compounds ZINC12151998, ZINC14970552, and ZINC11841871 were purchased from ChemBridge (San Diego, CA, USA).

### 3.2. LmTR Homology Model Development and Validation

The amino acid sequence of LmTR was obtained from the NCBI database (GenBank: XM_003871876.1). The search for sequential homologues to the amino acid sequence of LmTR was carried out by using the LiTR (2JK6) and TcTR (1BZL) crystal structures reported in database PDB/BLAST (http://blast.ncbi.nlm.nih.gov/Blast.cgi). The sequence alignment was performed using the Clustal-W program (available online: https://www.ebi.ac.uk/Tools/msa/clustalo/) and ENDscript server (available online: http://espript.ibcp.fr/ESPript/ESPript/) [30]. A three-dimensional model of LmTR was built using the SWISS-MODEL (Protein Structure Bioinformatics Group, Swiss Institute of Bioinformatics Biozentrum, University of Basel, Basel, Switzerland), I-Tasser (Department of Computational Medicine and Bioinformatics, Medical School, University of Michigan, Ann Arbor, MI, USA) and Phyre2 servers (Structural Bioinformatics Group, Department of Life Sciences, Imperial College London, London, UK) [31,32,33]. The model was evaluated by way of the Ramachandran plot, RMSD, and QMEAN4 score [20] to obtain an estimation of the local model quality, whereas the quality of the protein stereochemistry was assessed using the WHATCHECK server (European Molecular Biology Laboratory, Radboud University, Nijmegen, Gelderland, The NLD) [21].

The SWISS-MODEL was selected for further refinement and construction of the homodimer structure including NADPH and FAD according to crystallographic symmetry reports on LiTR. Finally, the homodimer structure model was subjected to energy minimization by employing the AMBER99SB-ILDN force field [34] and a single point charge water model with a time step of 0.002 ps using GROMACS 4.5.3 software (Department of Biophysical Chemistry of Groningen University, Groningen, Groningen, The NLD) [35]. 

### 3.3. Molecular Docking 

A subset of 20 druglike molecules with high affinity to LiTR (docking scores of −11.6 kcal/mol to −12.7 kcal/mol), obtained by virtual screening of 600,000 ZINC compounds [36], was previously reported [19]. In the present study their interaction with the catalytic site of LmTR was analyzed in silico. 

The structure of these 20 compounds was constructed and optimized using the Ligand Preparation module implemented in Maestro 9.6 [37]. The torsional root and branches of the ligands were chosen utilizing MGLTools 1.5.4 [38], allowing flexibility for all rotatable bonds. In addition, MGLTools 1.5.4 was used to assign Gasteiger–Marsili atomic charges to all ligands [39]. Docking calculations were performed using AutoDock 4.2 software (Department of Molecular Biology, La Jolla, CA, USA). A grid box of 60 × 60 × 60 points with grid spacing of 0.375 Å was calculated for the proper atom types and centered at the enzyme catalytic site. The Lamarckian genetic algorithm was used as a search method with a total of 10 runs being undertaken with a maximum number of 2,500,000 energy evaluations and initial populations of 150 conformers. The docking score for each compound was selected by considering their highest cluster size. To validate the LmTR model generated, the substrate trypanothione disulfide (T[S]_2_) and the TcTR inhibitor clomipramine were used.

### 3.4. Cloning, Expression, and Purification of Trypanothione Reductase from Leishmania mexicana

Genomic DNA from *L. mexicana* strain MNYC/BZ/62/M379 was used to amplify the LmTR gene by PCR. The forward oligonucleotide (5′-AGAAGAGAATTCATGTCCCGCTCCTACGA-3′) designed included an EcoRI restriction site (underlined) and the reverse oligonucleotide (5′-TCTTCTGCGGCCGCTTCAGAGGTTGCTGCTGAG-3′) included a NotI restriction site (underlined). The PCR product was cloned into PCR BLUNT II TOPO vector (Invitrogen) and sequenced. Then, it was subcloned into the expression vector pET28a plasmid (Novagen), which produced a protein with a His-tag in its N-terminal. 

BL21 (DE3) pLysS cells (Novagen) were transformed with pET28a-LmTR vector and grown in Luria-Bertani medium (500 mL) containing 50 µg/mL of kanamycin at 37 °C until the OD_600_ reached 0.5. After this, over-expression was induced with 1.0 mM isopropyl thio-β-d-galactoside (IPTG) for 16 h at 25 °C. Then, cells were harvested by centrifugation at 5000 rpm for a duration of 30 min and the pellet was washed twice with 100 mM Tris-HCl (pH 8.0). Cells were resuspended in lysis buffer (50 mM NaH2PO4, 300 mM NaCl, 10 mM imidazole, pH 8.0) containing the protease inhibitors N-*p*-Tosyl-L-phenylalanine chloromethyl ketone (TPCK) at 50 µg/mL (Sigma-Aldrich, St. Louis, MO, USA) and Phenylmethylsulfonyl fluoride (PMSF) at 200 µM (Sigma-Aldrich) and lysed by sonication. After centrifugation, the supernatant was passed through a Ni-NTA affinity column and the recombinant protein (rLmTR) was purified with a gradient of 50–250 mM imidazole. The protein concentration was determined by the Bradford method.

Purified protein (20 µg) was electrophoretically separated by 12% SDS-PAGE at 100 V for 1 h 30 min. The gel was Coomassie blue stained to assess the presence and quality of purified protein. To confirm the dimeric nature of rLmTR, 7.5% native-PAGE in Tris–glycine buffer, pH 8.6, was run following the conditions previously reported [40]. Bovine serum albumin (60 kDa) and lactate dehydrogenase (140 kDa) were included as molecular weight markers. 

### 3.5. Biochemical Characterization of rLmTR

The enzymatic activity of rLmTR was assayed spectrophotometrically as previously described [41]. The reaction mixture contained 40 mM Hepes, pH 7.5, 1 mM EDTA, 150 µM NADPH, and 30 ng rLmTR. The reaction was initiated by adding the substrate (T[S]_2_) at 400 µM and was allowed to proceed for 1 min. NADPH oxidation was followed at 340 nm, monitored every 20 s, in a Synergy™ H1 microplate reader (Biotek Instruments, Inc. Winooski, VT, USA). The decrease in the absorbance at 340 nm was used to determine the moles of substrate consumed. One unit of enzyme activity is defined as the amount of enzyme required to convert 1 μmol of NADPH to NADP per minute at 25 °C [42].

The Km value of rLmTR was determined at 150 µM NADPH and different concentrations of T[S]_2_ (20, 50, 100, 150, 250, 400, and 600 µM). The kinetic data were analyzed by linear regression fit to Michaelis–Menten kinetics and linearized by the double reciprocal transformation of Lineweaver and Burk [43]. 

### 3.6. Inhibition of rLmTR Activity

Compounds ZINC04684558, ZINC09642432, ZINC12151998, ZINC14970552, and ZINC11841871 with affinity to the LmTR catalytic site with docking scores of at most −5.29 kcal/mol were selected to evaluate their in vitro inhibition of rLmTR activity. Due to their solubility, the inhibitory activity of compounds was tested at different concentrations dissolved in DMSO at 10% final concentration. The assay was carried out following the conditions previously described to determine the rLmTR activity. The percentage of inhibition was obtained using the following equation:

(1) $$\%\;\mathrm{Inhibition}=\frac{A_{0}-A_{1}}{A_{0}}\;-\;100$$

where A_0_ is the enzyme activity without inhibitor and A_1_ is the enzyme activity in the presence of inhibitor.

### 3.7. Molecular Dynamic Studies 

Two different conditions were used for molecular dynamics studies (MD) of the enzyme–inhibitor complexes. In the first condition, in order to determine the stability of the homodimer, a simulation of the LmTR model including NADPH and FAD was carried out. In the second instance, the stability of the enzyme–inhibitor complexes was analyzed. It is important to highlight that the enzyme with the inhibitor was modeled in both active sites (1 and 2) of the homodimeric enzyme using the docking methodology mentioned. The complexes obtained were used for the molecular dynamics simulations runs. Ligand parameters were calculated in a PRODRG server (Centre for Gene Regulation and Expression, School of Life Sciences, University of Dundee, Scotland, UK) [44] with a GROMOS87 force field (Biomos b.v., Laboratory of Physical Chemistry, Zürich, Switzerland) [45]. All simulations were carried out using the GROMOS9643a1 force field [35]. To perform molecular dynamics simulations, the homodimeric protein and the protein–inhibitor complexes were prepared at the beginning by an energy minimization process using 500 cycles of the steepest descent algorithm. The first velocities were assigned according to the Maxwell distribution to a temperature of 10 K, and then gradually increased to 300 K. After that, canonical (NVT) [46] and isothermal–isobaric (NPT) [47] simulations (with isotropic position scaling) were carried out at 300 K and 1 atm pressure using a truncated cubic periodic box with dimensions of 100 Å, filled with a TIP3P water model and neutralized with Na^+^ and Cl^−^ ions. Final simulation runs were carried out for 50 ns at 300 K without restrictions, obtaining 5000 conformations that were saved every 5000 steps.

### 3.8. ADME Characterization of Selected LmTR Inhibitors 

The Faf-Drugs4 server (Université Paris Diderot, Paris, FRA) [48] and the Molsoft L.L.C. (available online: http://molsoft.com/mprop) were used for calculating relevant druglike properties, and Data Warrior software (Actelion Pharmaceuticals Ltd, Allschwil, Basel, Switzerland) [49] was used to determine mutagenesis and tumorigenicity. For the prediction of the toxicological parameters and the LD_50_, the PROTOX server [50] was used.

### 3.9. Activity Evaluation against Leishmania mexicana Promastigote 

The in vitro activity of compounds ZINC04684558, ZINC09642432, ZINC12151998, ZINC14970552, and ZINC11841871 was evaluated against promastigotes of *L. mexicana* strain MNYC/BZ/62/M379 using the resazurin method previously described [51]. For this, 5 × 10^5^ parasites/well were cultured in 96-well microliter plates, and compounds were dissolved in DMSO and diluted in the culture medium at concentrations ranging from 100 to 0.8 µg/mL in a final volume of 200 µL. Amphotericin B, miltefosine, and glucantime were used as the reference drugs. After incubation for 48 h at 26 °C, 20 µL of 2.5 mM resazurin solution was added to each well and incubated for 4 h. The fluorescence intensity (544 nm excitation wavelength and 590 nm emission wavelength) was measured using a fluorometer (Thermo Scientific Fluoroskan Ascent byThermo Fisher Scientific, Waltham, MA, USA). All assays were carried out in triplicate. The half-maximal inhibitory concentration (IC_50_) was determined by Probit analysis.

### 3.10. Macrophage Cytotoxicity Assays

The cytotoxicity of the five selected compounds was tested using murine macrophage cell line J774.2 (ATCC ^®^TIB-67). Amphotericin B, miltefosine, and glucantime were included as reference drugs. For this, macrophages were seeded (5 × 10^4^ cells/well) in 96-well flat-bottomed microplates and allowed to adhere for 24 h at 37 °C in 5% CO_2_. The culture medium was then replaced with different concentrations of the compounds (100 to 0.8 µg/mL), followed by incubation for another 24 h. All assays were carried out in triplicate. Thereafter, 20 µL of a 2.5 mM resazurin solution was added to each well, and the plates were incubated for another 4 h. The fluorescence emission was measured as described above. The half-maximal cytotoxicity concentration (CC_50_) was determined by Probit analysis.

## 4. Conclusions

This study achieved the first characterization of the inhibition of rLmTR activity by five compounds found in the ZINC database with affinity to the LmTR catalytic site (docking scores −10.27 kcal/mol to −5.29 kcal/mol). The inhibition of rLmTR was demonstrated to be in the range of 32.9% to 40.1%. All compounds showed higher leishmanicidal activity than glucantime. In particular, compound ZINC12151998 (docking score −10.27 kcal/mol) inhibited 32.9% the enzyme activity (100 µM). It showed the highest leishmanicidal activity (IC_50_ = 58 µM) of all the selected compounds, and it was more active than glucantime (IC_50_ > 273.2µM). Although its cytotoxicity (CC_50_ = 53 µM) was higher than that of the other four compounds, it was less cytotoxic than amphotericin B. Compound ZINC12151998 is an *N*-(6-quinolinemethyl)-3-pyrazole carboxamide, and the participation of the quinoline nucleus in the formation of hydrogen bonds with the active site of LmTR was shown by molecular dynamics studies.

Therefore, compound ZINC12151998 provides a promising starting point for a hit-to-lead process in our search for new anti-*L. mexicana* drugs that are more potent and less cytotoxic. 

## Figures and Tables

**Table 1:** *Leishmania mexicana* trypanothione reductase (LmTR) models constructed through homology modeling.

Server	RMSD *	Q-MEAN4 **	Ramachandran Plot (%)	
			F	P
SWISS-MODEL	0.063	−0.17	96.4%	99.7%
I-Tasser	0.257	−3.34	89.0%	96.9%
Phyre2	0.269	−1.20	91.51%	99.8%

Note: Ramachandran plot regions: (F) residues in most favored regions and (P) residues in additional allowed regions. * Root-mean-square deviation. ** Qualitative Model Energy Analysis

**Table 2:** Inhibition of rLmTR activity by selected compounds.

Compounds	% Inhibition
ZINC14970552 (300 µM)	35.7
ZINC09642432 (300 µM)	40.1
ZINC04684558 (300 µM)	39.5
ZINC11841871 (150 µM)	39.5
ZINC12151998 (100 µM)	32.9

**Table 3:** Hydrogen bonding interactions in LmTR–inhibitor complexes at different times throughout the molecular dynamics simulations.

Compound		Time (ns)					
	Active Site	0	10	20	30	40	50
ZINC14970552	1	A: Met333	-	A: Tyr198	-	-	A: Tyr198
	2	B: Glu18	-	-	-	-	-
ZINC09642432	1	-	-	-	-	-	-
	2	-	B: Leu167 B: Gly168	B: Leu167	-	-	B: Leu167
ZINC04684558	1	A: Tyr110 B: His461	B: Asn402	-	-	-	-
	2	B: Val58	-	B: Ser109	B: Ser109	B: Ser109	B: Ser109 A: Glu466
ZINC11841871	1	B: His461	B: Ser470 B: His461	A: Val58	A: Val58	A: Ser14	B: Arg472
	2	B: Tyr110	B: Tyr110	B: Tyr110	-	-	-
ZINC12151998	1	-	B: Gly459	-	-	-	A: Arg355
	2	-	-	B: Tyr110 A: Glu467	B: Tyr110	B: Tyr110 A: Glu466 A: His461	B: Tyr110

**Table 4:** Physicochemical and toxicological profiles of LmTR inhibitors.

Molecule	MW ^a^	HBD ^a^	HBA ^a^	LogP ^a^	LD_50_^b^ (mg/kg)	Toxicity Class ^b^	Toxicity Targets ^b^	Mutagenicity ^c^	Tumorigenicity ^c^	Drug Likeness ^d^
ZINC11841871	494.5	7	2	3.6	1000	4	none	none	none	0.68
ZINC12151998	486.5	7	1	4.8	500	4	none	high	none	0.76
ZINC14970552	443.5	7	1	3.7	1000	4	none	none	none	1.09
ZINC04684558	409.3	6	2	4.9	1600	4	none	none	none	0.08
ZINC09642432	463.4	8	1	2.9	1870	4	none	none	none	0.69

^a^ Server FAFDrugs, filter druglike soft was used: MW 100–600; HBD ≤ 5; HBA ≤ 12; log P −3 to 6. ^b^ Toxicity class was determined in Server PROTOX; values ranged between 1 and 6 where 1 is toxic and 6 is safe. Toxicity targets were determined for adenosine A2A receptor, adrenergic beta 2 receptor, androgen receptor, amine oxidase, dopamine D3 receptor, estrogen receptor 1 and 2, glucocorticoid receptor, histamine H1 receptor, nuclear receptor subfamily 1 group I member 2, opioid receptor kappa, opioid receptor mu, cAMP-specific 30,50 -cyclic phosphodiesterase 4D, prostaglandin G/H synthase 1, progesterone receptor. ^c^ Mutagenic and tumorigenic effects were determined using Data Warrior. ^d^ Drug likeness score was determined with Server Molsolf; values between −1 and 2 were accepted.

**Table 5:** Leishmanicidal activity, cytotoxicity, and selectivity index of compounds from ZINC database.

Compounds	IC_50_ (µM)	CC_50_ (µM)	SI *
ZINC04684558	263	338	1.2
ZINC12151998	58	53	0.9
ZINC14970552	147	219	1.4
ZINC09642432	222	335	1.5
ZINC11841871	159	103	0.6
Glucantime	>273.2	>273.2	1.0
Amphotericin B	0.11	7.4	67
Miltefosine	2.98	141	47

* The selectivity index (SI) values of ZINC compounds were calculated as the ratio of cytotoxicity to biological activity (SI = CC_50_/IC_50_).

## Supplementary Materials

Supplementary materials are available online. Figure S1: LmTR in complex with ZINC14970552 (**a**), ZINC09642432 (**b**), ZINC04684558 (**c**), ZINC11841871 (**d**), and ZINC12151998 (**e**).
