# Supplementary material for: Leishmania mexicana Trypanothione Reductase Inhibitors: Computational and Biological Studies
Source: Molecules. 2019 Sep 4;24(18):3216. doi: 10.3390/molecules24183216 (PMC6767256; doi:10.3390/molecules24183216)
Supplement: Supplementary file 1 [file molecules-24-03216-s001.pdf]

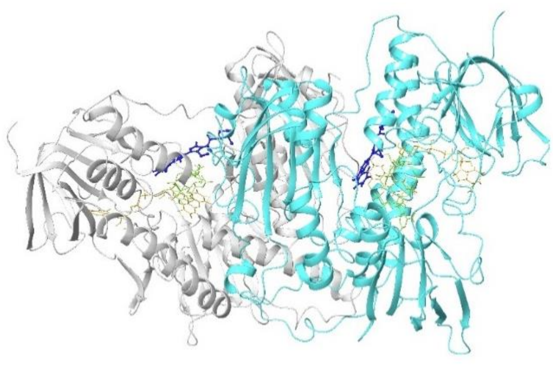

(a)

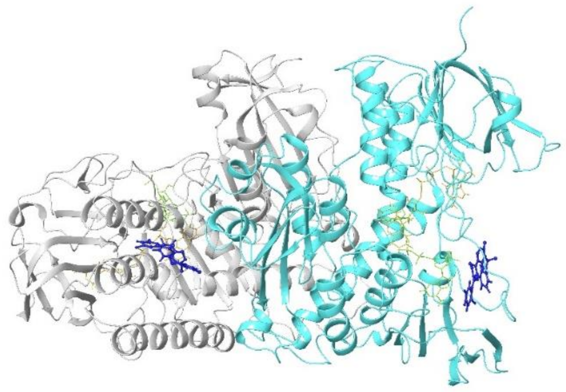

(b)

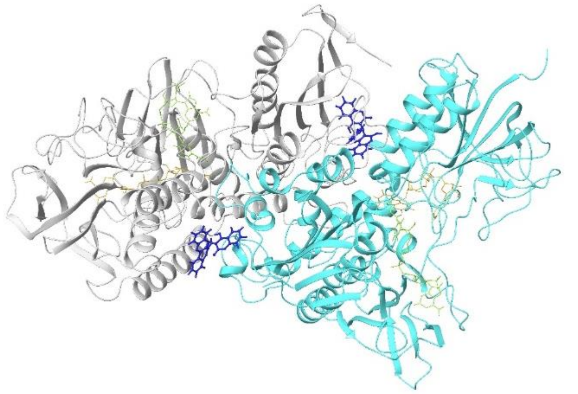

(c)

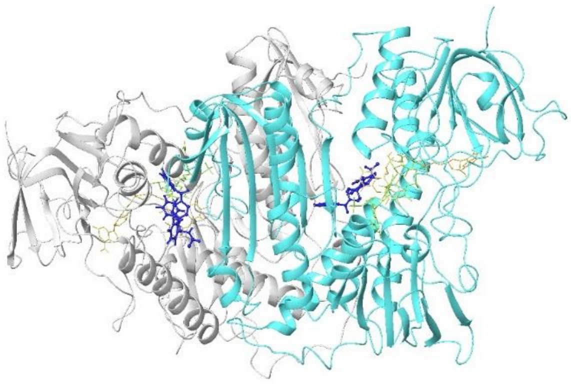

(d)

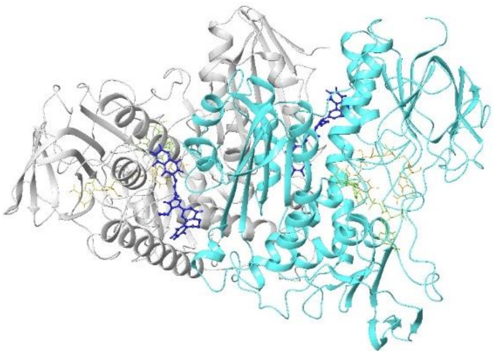

(e)

Figure S1. LmTR in complex with ZINC14970552 (a), ZINC09642432 (b), ZINC04684558 (c), ZINC11841871 (d) and ZINC12151998 (e). The LmTR monomers A and B are represented with gray ribbons and cyan ribbons, respectively. The ligands are represented in blue sticks FAD and NADH are represented in thin sticks yellow and green, respectively.
